# Age-related diseases as a testbed for anti-aging therapeutics: the case of idiopathic pulmonary fibrosis

**DOI:** 10.18632/aging.206301

**Published:** 2025-08-16

**Authors:** Alex Zhavoronkov, Dominika Wilczok, Feng Ren, Fedor Galkin

**Affiliations:** 1 Insilico Medicine AI Limited, Abu Dhabi, UAE; 2 Insilico Medicine US, Inc., Boston, MA 02138, USA; 3 Insilico Medicine Hong Kong Ltd., Hong Kong Science and Technology Park, Hong Kong SAR, China; 4 Buck Institute for Research on Aging, Novato, CA 94945, USA; 5 Duke University, Durham, NC 27708, USA; 6 Insilico Medicine Shanghai Ltd., Shanghai, China

**Keywords:** aging, drug development, idiopathic pulmonary fibrosis, ipf, scoring

## Abstract

While aging-related diseases are often used as proxies for the general aging process in research, they largely differ in terms of how integral their development is to organismal aging. Hallmarks of aging offer a convenient conceptual framework to assess the relevance of a disease to the anti-aging narrative. In this review, we propose hallmark decomposition as a method of measuring the potential of a therapy to translate from a disease-specific treatment to a general geroprotector. To help other researchers adopt and improve this methodology, we are releasing a disease scoring system and report the hallmark alignment of 13 aging-related diseases, among which IPF is the disease most aligned with aging.

## The hallmarks of aging: a framework for understanding disease

Hallmarks of aging as a concept represent the failure of the biologists’ earlier attempts to define a singular driver of aging. Mechanistic theories explaining aging as an evolutionary program or a mitochondrial dysfunction, or any other key source of degeneration could not explain the aging phenotype in its completeness [1–3]. Hence an alternative was proposed in defining aging as a combination of these various mechanisms chipping away at an organism as the “hallmarks of aging” [4].

A slightly predating concept of the “hallmarks of cancer” suggested that the development of malignant tumors rests on a set of primary enabling processes, such as somatic mutations, which eventually give way to emergent, secondary processes that result in the malignancy’s growth [5]. Similarly, the hallmarks of aging are understood as a triad of primary, antagonistic (compensatory), and integrative processes immediately linked to the aging phenotype. The critics of the aging hallmarks concept indicate, however, that the aging-cancer analogy is not complete and some key distinctions are often overlooked [6]. In particular, genomic instability is the primary cause of all tumors while its aging analog, cellular damage, is a term that is much more open to interpretation and debate [6]. Another point of criticism is what constitutes a hallmark and how many of them there are. While the original 2013 publication defined nine processes, the most recent review of the framework features 12 of them [7–9]. The partition between primary and secondary aging hallmarks is not as clear as in cancer and the boundary between two hallmarks is often diffused, as is the case with inflammation which can hardly be isolated from other hallmarks [10].

Despite their theoretical drawbacks, the hallmarks of aging have been widely adopted both in academia and the biotechnology industry as a framework for their practical utility. They offer a language to describe the proxies of aging used in research. As pointed out by the late Leonard Hayflick, the undefined terminology is the bane of biogerontology leading to “communication failures, erroneous interpretations, and misdirected decisions” [11, 12]. The hallmarks framework allows the anti-aging efforts to be more specific in what type of aging is accelerated or prevented, and thus, provides a more reliable way to find the applications for the observed anti-aging effects.

The hallmarks also give us an opportunity to explore the bidirectional and complex connection between aging and its manifestations in diseases. While aging reduces the overall resilience of an organism, certain pathologies exhibit particularly strong associations with specific aging hallmarks, making them valuable models for investigating geroprotective strategies.

**Figure 1:**
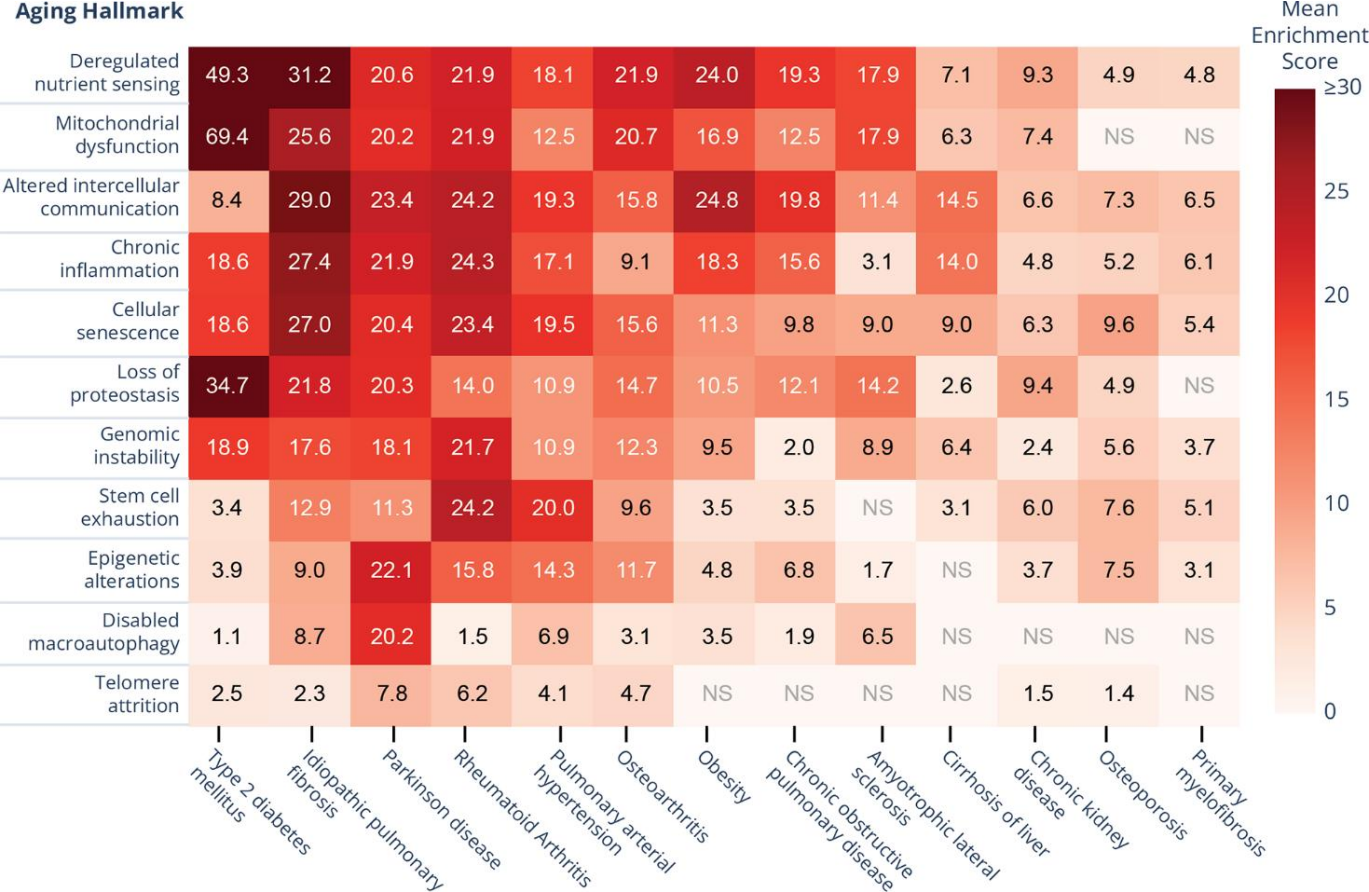
**Heatmap representation of hallmark scores across 13 aging-related diseases (ARDs).** Higher numbers mean higher abundance of hallmark-specific genes among an ARD's targets. Among the scored diseases, type-2 diabetes and idiopathic pulmonary fibrosis demonstrate the strongest representation across multiple hallmarks, particularly in deregulated nutrient sensing. This analysis reveals distinct patterns of hallmark involvement across different ARDs, with some hallmarks (mitochondrial dysfunction, chronic inflammation) broadly represented across multiple conditions, while others (e.g. disabled macroautophagy) show more selective disease associations. NS: Not Significant at q-value<0.001 after Benjamin-Hochberg correction; mean enrichment scores calculated based on 250 samples of target genes for each disease; the plot is produced in Plotly 5.23 for Python 3.11.

This perspective explores how aging-related diseases (ARDs) can serve as experimental platforms for discovering new geroprotective interventions. We place special emphasis on idiopathic pulmonary fibrosis (IPF) as an emblematic ARD based on extensive literature evidence, findings in preclinical and clinical studies, and a hallmark deconvolution scoring (Figure 1).

The scoring is based on the hallmark-associated pathway enrichment in the published targets associated with a disease (see Methods). We propose to extend the concept of the hallmarks of aging by making it more practically inclined and relevant to anti-aging therapeutics. By deconvoluting the conditions as a sum of aging-related processes, we suggest a measure of biogerontological relevance that may be applied to clinical trials and case-control studies. Furthermore, this measure can make biogerontology more “empirically progressive” by serving as a criterion for a therapy’s geroprotector potential [13]. If the effectiveness of a target, mechanism of action, or compound increases proportionally with how relevant a particular setting is to biological aging, then this entity is more likely to also have positive effects on aging itself, rather than only the indicated disease. In other words, we suggest that clinical efficacy against ARDs should be considered a necessary criterion for validating putative geroprotectors, with IPF representing a particularly informative testbed due to its strong association with multiple aging hallmarks.

## Mechanistic clustering of ARDs

The hallmarks framework is commonly used to assess the relevance of a model, a disease, intervention, or a phenotype to the aging process [14–16]. Pathologies whose etiology is linked to the hallmark molecular mechanisms are described as aging-related. Such diseases are typically non-communicable, characterized by chronic development, may manifest after a long asymptomatic period, and often involve progressive functional decline. Some researchers propose a more precise definition of an ARD based on epidemiologic data indicating a higher incidence in older age groups [17]. But for research purposes, the aging-related status is most commonly established from a mechanistic perspective.

Despite the fuzzy definition, some ARDs may be clustered together based on which hallmarks play the key role in their development [18]. One such cluster are inflammatory aging diseases, which include rheumatoid arthritis, chronic kidney disease, diabetes, inflammatory bowel disease, and others [19]. These diseases are characterized by a chronic low-grade inflammation, immune cell senescence, and dysregulation of innate and adaptive immune responses. This array of phenomena, also called “inflammaging”, manifests as elevated levels of pro-inflammatory cytokines such as *IL-1β*, *IL-6*, and *TNF-α*, which create a tissue environment conducive to disease progression [20]. The relationship between inflammation and aging is complex and bidirectional. While inflammatory processes contribute to tissue damage, they may also represent adaptive responses to accumulating stressors. This duality is exemplified by centenarians, who paradoxically exhibit elevated levels of pro-inflammatory cytokines alongside enhanced anti-inflammatory mechanisms that maintain homeostasis [21, 22]. The case of *IL-6* illustrates this complexity: while its inhibition shows therapeutic potential in conditions like rheumatoid arthritis and potentially IPF, *IL-6* also plays essential roles in tissue regeneration and metabolism [23].

Another ARD cluster defined based on its driver hallmark are metabolic diseases, which include non-alcoholic fatty liver diseases (NAFLD), metabolic syndrome, atherosclerosis, and diabetes [24, 25]. Deregulated nutrient sensing, particularly insulin resistance, serves as a central pathogenic mechanism in all these conditions [26]. This deregulation is closely linked to other hallmarks, such as mitochondrial dysfunction, inflammation, and the accumulation of senescent cells, which collectively exacerbate metabolic dysfunction [27–29]. These interacting hallmarks create a complex pathogenic network that becomes increasingly difficult to disrupt with age.

## Anatomical clustering and limitations

While clustering ARDs by predominant hallmarks provides valuable mechanistic insights, the complex interplay between these hallmarks reveals the limitations of such classification. The hallmarks of aging rarely operate in isolation, blurring the boundaries between the clusters. An alternative approach to ARD classification focuses on the primarily affected organ systems, rather than the underlying mechanisms. This anatomical perspective yields clusters such as neurodegenerative diseases, which encompass conditions like Alzheimer's disease, Parkinson's disease, and amyotrophic lateral sclerosis. While these diseases share the common feature of progressive nervous system deterioration, they emerge from diverse combinations of aging hallmarks.

Neurodegenerative diseases exemplify how multiple aging hallmarks converge to drive pathogenesis. In Alzheimer's disease, proteostasis failure manifests as β-amyloid plaques and tau neurofibrillary tangles, while altered intercellular communication disrupts synaptic function [30]. Mitochondrial dysfunction contributes through reduced ATP production and elevated reactive oxygen species in cortical neurons [31]. Cellular senescence further exacerbates these pathologies, with senescent astrocytes and microglia releasing pro-inflammatory factors that accelerate neurodegeneration [32]. The interplay between these hallmarks creates a vicious cycle of neuronal damage that ultimately leads to cognitive decline.

These classification approaches provide useful frameworks but remain inherently subjective. The complex molecular networks underlying ARDs resist simplistic categorization, with most diseases involving multiple hallmarks across various tissues. This complexity highlights the need for quantitative methods to assess the relationship between specific diseases and aging biology. We propose conceptualizing ARDs along a spectrum based on their alignment with aging biology. At one end are conditions only tangentially related to aging, where age serves primarily as a risk factor rather than a central pathogenic mechanism. At the other end are diseases so inextricably linked to aging processes that they essentially represent accelerated or localized manifestations of aging itself. In the following section, we demonstrate a way to construct such a spectrum to identify the diseases that would serve as optimal testbeds for geroprotective interventions.

## Aging alignment in pathologies

We propose organizing ARDs based on how aligned they are with the wider aging process based on their clinically relevant target genes. We have chosen to annotate 13 ARDs discussed in detail in [33] to identify the disease that is the most similar to the aging process. To achieve this, we scored each disease based on the participation of disease targets in hallmark-associated pathways. The score was calculated based on the results from enrichment analysis of the targets with a hallmark-annotated version of Gene Ontology Biological Processes (GO:BP) ontology with an adjustment rewarding hallmark diversity (see Methods). This method allowed us to identify the diseases whose patients were most likely to benefit from geroprotector discovery efforts, and conversely, which indications would make a better testbed for anti-aging research.

From a wider perspective, our scoring reveals distinct hallmark co-occurrence patterns across ARDs, suggesting that these aging mechanisms are fundamentally interconnected with multiple pathological manifestations (Figure 1). For instance, deregulated nutrient sensing emerges as the most broadly represented hallmark, with high scores spanning from metabolic diseases like type 2 diabetes (49.3 out of 100) to fibrotic conditions like IPF (31.2) and inflammatory disorders like osteoarthritis (21.9). Such cross-disease hallmark sharing suggests that therapies addressing metabolic dysfunction could benefit diverse ARDs beyond their primary indication.

**Figure 2:**
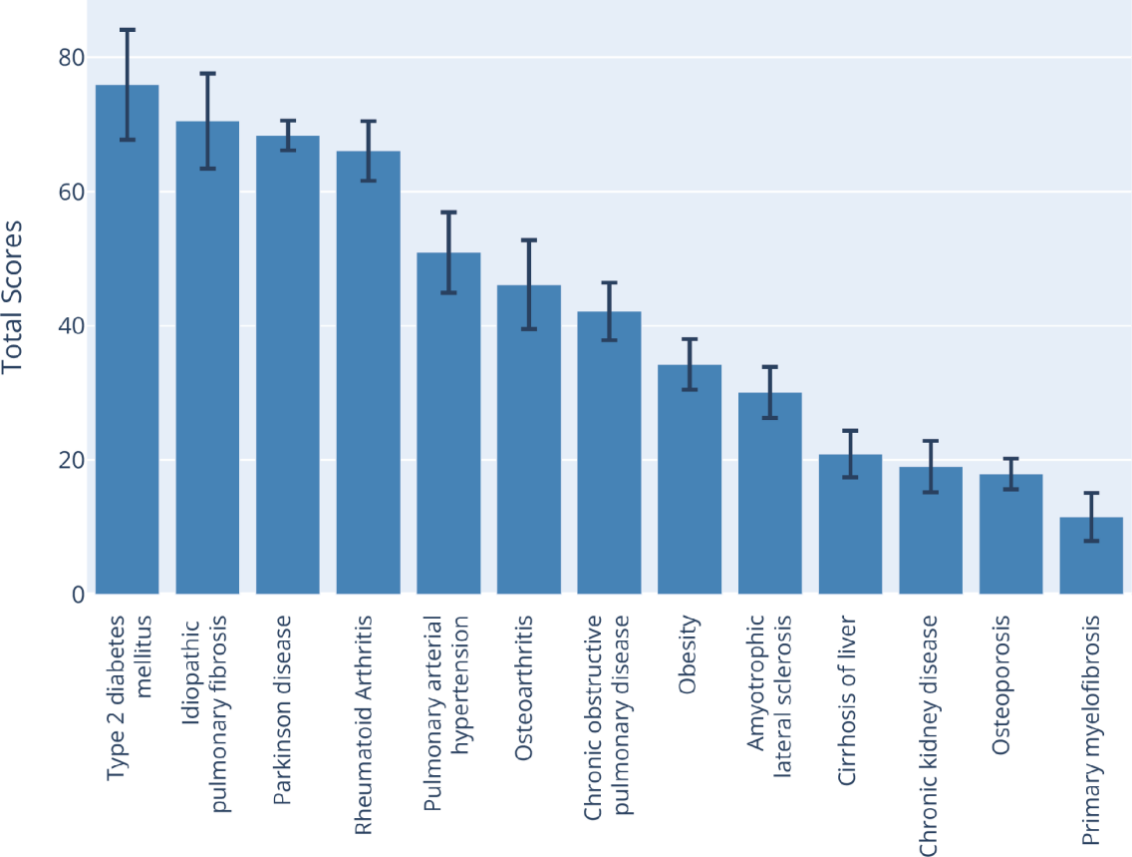
**Quantitative comparison of aging alignment scores across 13 aging-related diseases.** The total score represents the similarity of a pathology to the general aging process, calculated as a weighted sum of partial hallmark scores (see Methods). Type-2 diabetes, idiopathic pulmonary fibrosis, Parkinson’s diseases, and rheumatoid arthritis demonstrate substantially higher alignment with aging processes compared to other conditions. This analysis supports the hypothesis that IPF is an optimal testbed for geroprotective interventions due to its strong mechanistic overlap with fundamental aging processes. Bar height represents the mean sampling score, whiskers display the standard deviation, based on the sampling procedure described in Methods; the plot is produced in Plotly 5.23 for Python 3.11.

This multi-hallmark principle is exemplified by the highest-scoring ARDs, which include type 2 diabetes, IPF, Parkinson's disease, and rheumatoid arthritis (all >60 out of 100; Figure 2). These diseases demonstrate substantial involvement across multiple hallmarks simultaneously: primarily, deregulated nutrient sensing, altered intercellular communication, chronic inflammation, cellular senescence. Consequently, preclinical interventions addressing such prevalent hallmarks may achieve superior therapeutic outcomes across all high-scoring ARDs, making them equally valuable testbeds for geroprotector discovery. Among other indications, IPF stands out within due to its rapid progression and well-defined clinical endpoints, offering practical advantages for testing anti-aging interventions.

The scoring also reveals therapeutically underexplored areas that represent a double-edged strategic opportunity. While hallmarks like deregulated nutrient sensing and mitochondrial dysfunction show broad disease representation with numerous known targets, others such as telomere attrition and disabled macroautophagy, exhibit more sparse associations. This pattern likely reflects differential target discovery investment rather than biological irrelevance, as telomere attrition remains a well-documented contributor to multiple ARDs [34]. For sparse hallmarks, fewer known targets imply higher discovery risk, but also lower competition and potentially greater impact for successful interventions. The comparison of hallmarks' contribution across the ARD spectrum may help drug developers strategically choose between targeting crowded, well-validated pathways or exploiting underexplored therapeutic niches [35].

While some authors express skepticism about the value of hallmarks in drug discovery, highlighting issues such as conflating biomarkers and drivers of aging or individual hallmarks' fuzzy definition [6, 36, 37], the framework has generated a fruitful research program that now warrants empirical validation through intensive clinical testing [38]. To further this point, some broad anti-aging strategies, such as senolytics, have been shown to lead to improve the outcomes in Alzheimer’s disease, ischemic injury, diabetes and other indications [39–43]. Such evidence validates the translational potential of hallmark-first drug development.

We argue that aging biology should be considered early in the drug design process, rather than addressed only later through repurposing efforts. The deconvolution of disease-hallmark interactions proposes a more flexible and robust alternative to the disease-centric mode of drug development. The specific approach presented here should be treated as one of many possible options for disease-hallmark annotation, with future improvements possible through additional data sources and methodological refinements.

## Idiopathic pulmonary fibrosis: the archetypal aging disease

Despite its potential improvements, the presented metric of an ARD’s alignment with the general aging process can be used to extract useful insights for anti-aging therapeutics. More specifically, our method has identified IPF as one of the top-scoring ARDs, which is backed by the latest literature describing IPF as an exemplary convergence of aging biology and clinical disease [44].

IPF is a chronic, progressive interstitial lung disease characterized by relentless scarring of lung tissue, declining pulmonary function, and a life expectancy of 3-4 years post-diagnosis [45, 46]. While normal aging is associated with a decline of 13-34 mL/year in the lung’s forced vital capacity of older adults, in untreated IPF patients the rate of decline reaches 130-210 mL/year [47, 48]. The existing therapies for IPF are designed to grant the patients enough time to obtain a lung transplant, the only treatment that meaningfully extends their lifespan to 6 years post-operation [49, 50]. The severity and the progression rate of this disease make the lack of restorative treatments a particularly pressing issue.

The incidence of IPF rises sharply with age, satisfying the epidemiological definition for ARDs [51]. In addition, IPF is driven by practically all hallmarks of aging jointly contributing to its debilitating effects on the patient [44]. This intersection of clinical urgency and high aging alignment makes IPF a uniquely attractive platform for testing anti-aging interventions.

The rich hallmark interaction network sustaining IPF progression presents multiple attack vectors for an aging-focused therapy. High genomic instability in alveolar epithelial cells and fibroblasts is a major contributor to IPF progression, as shown in a recent work on the somatic mutation load in IPF patients [52, 53]. Other studies show that 10-20% of familial IPF patients carry mutations in telomerase maintenance genes (*TERT*, *TERC*, *RTEL1*), as well as other hazardous variants in oncogenes (*TP53*), cytokine (*TGF-β*, *IL-8*), and matrix remodeling (*MMP1*) genes [54, 55]. The correlation seen in the mutation landscapes of IPF and lung cancer has led some authors to hypothesize that lung cancer and IPF have a shared etiology [54]. In this context, it is not surprising to see that nintedanib, a drug approved for IPF treatment in 2014, was originally discovered as a solid tumor treatment [56].

Other instances of hallmark-aligned pathogenic processes in IPF include aberrant epigenetic regulation by histone deacetylases (HDACs), including sirtuins, that is suspected to contribute to such IPF milestones as epithelial cell death and mesenchymal transition, fibroblast persistence, ECM deposition, and others [57]. Moreover, lung samples from IPF patients showed higher biological age compared to healthy controls using four different DNA methylation clocks [58]. Some teams argue that HDAC-inhibitors used in oncology may be repurposed to treat IPF [59]. Additionally, some selective sirtuin activators discussed in longevity research, such as resveratrol and calorie restriction, may be able to normalize epigenetic regulation in IPF [57, 60]. The clinical potential of calorie restriction in IPF treatment is particularly intriguing due to its status as a highly validated life extension method [61]. In this light, the shortage of nutritional studies in IPF patients is a major setback for both clinical and anti-aging research [62]. For an in-depth review of other hallmarks manifesting in IPF, we encourage the reader to read the recent in-depth review [44].

## Anti-aging IPF therapeutics

Anti-IPF drug development has become a highly competitive field with over 100 phase-2 and -3 trials registered over the last 10 years [63]. With this many clinical programs nearing their resolution, the approval criteria for new drugs are bound to become stricter. Novel treatments would be required to hold advantage over existing standard-of-care options, which implies a shift from drugs stalling IPF progression to restorative therapies. This impeding shift stimulates the adoption of new methodologies and discovery processes, including AI-assisted approaches [64]. The pressure to innovate has already led multiple research teams and pharmaceutical companies to base their drug design strategy on the concept of aging hallmarks (Table 1) [65].

**Table 1:** Overview of emerging IPF therapeutic strategies that affect hallmarks of aging.

**Drug**	**Mechanism of action (aging hallmark affected)**	**Current status in trials**	**Trials organized by (country)**
BI 765423 [66, 67]	Anti-IL-11 antibody, reduces fibroblast activation (inflammaging)	Phase-1 completed	Boehringer Ingelheim (Germany)
Rentosertib (ISM018_055) [33, 64, 83]	TNIK inhibitor, modulates Wnt/β-catenin signaling, reducing fibroblast activation and epithelial senescence (nutrient sensing, ECM stiffness, genomic instability, stem cell exhaustion, altered intercellular communication, and cellular senescence)	Phase-2a completed, leads to forced vital capacity increase, identified senomorphic properties	Insilico Medicine (China, US, UAE)
Dasatinib + Quercetin [75, 77]	Senolytic combination (cellular senescence)	Phase-1 pilot trial in IPF completed	Wake Forest School of Medicine (US)
BCL-inhibitors [78]	Apoptosis induction in senescent cells and activated fibroblasts (cellular senescence)	Preclinical IPF models	Unity Biotechnology (US)
Danazol [98, 100, 101]	Synthetic androgen that upregulates TERT (telomere attrition)	Phase-2 ongoing	University of Queensland (Australia)
Telomerase Gene Therapy (AAV-TERT) [97]	Gene therapy delivering TERT via AAV to lung cells (telomere attrition)	Promising result in murine models	Telomere Therapeutics (Spain)
Ziritaxestat (GLPG1690) [87, 88]	Autotaxin pathway inhibition (unknown)	Phase-3 discontinued	Galapagos NV, Gilead Sciences (Belgium, US)
Cycloastragenol (GRN510, TA-65) [90, 92]	TERT activator extracted from Astragalus (primarily telomere attrition)	Pilot trials in humans	Telomerase Activation Sciences (US)
Resveratrol, calorie restriction… [57]	Sirtuin activation, HDAC inhibition (epigenetic modulation, nutrient sensing)	Hypothesis formation stage	-

Hallmarks of aging play a major role in the onset and development of IPF, making hallmark-focused drug development a promising strategy. As these drug candidates move along the clinical pipeline, their potential as geroprotectors also increases due to the similarities IPF and the general aging process share.

Some insights from aging research are already translating into drug development, with several therapeutic strategies targeting IPF while concurrently modulating fundamental aging mechanisms. BI-765423 is an anti-IL-11 antibody undergoing a phase-1 trial (NCT05658107) [66, 67]. This treatment targets an important participant of the inflammaging process, whose inhibition has been shown to extend murine lifespan, counteract senescent transformation in human cells, and reduce fibroblast activation [68, 69].

Targeting senescence has proven to be a powerful strategy to integrate anti-aging and anti-IPF treatments [70]. According to some authors, primary lung epithelium senescence caused by prolonged cell damage is the root cause responsible for the onset of IPF [71]. As they elaborate further, the chemokines secreted by the senescent epithelium promote secondary senescence in fibroblasts and myofibroblasts, whose secretome, rich in *MMP12*, *IL-6*, *TGF-β,* actively promotes inflammation and ECM remodeling [72, 73]. The driving role of senescence in IPF pathogenesis has led to multiple *in vitro*, *in vivo*, and even human pilot experiments featuring senolytics [74, 75]. The dasatinib+quercitin intermittent combination is particularly promising due to three factors: the molecular target similarity to an approved drug (nintedanib), lack of severe side effects, and the promising results in the models of other ARDs [76, 77].

In preclinical mouse models, the ABT-263 (navitoclax) senolytic inhibits antiapoptotic *BCL-2*, selectively inducing apoptosis in profibrotic fibroblasts and reducing lung collagen deposition [78]. Unity Biotechnology, in collaboration with the Buck Institute for Research on Aging, has patented a series of inhibitors of the BCL protein family, which show promise in treating IPF by selectively targeting and eliminating senescent cells in lung tissue, as supported by preclinical screening data [79]. Although the IPF patent has been abandoned, Unity Biotechnology continues exploring senolytics’ applications in other fibrotic diseases [80].

Another powerful strategy to combat both aging and IPF could be preventing senescent cell formation rather than destroying them with senolytics. A novel phase-2 (NCT05975983) IPF drug candidate, rentosertib, shows a reduction of senescent cell formation in a replicative senescence model, which could contribute to its clinical efficacy [81–83]. Rentosertib’s anti-aging properties have additionally been validated by a reduction in pro-inflammatory and pro-fibrotic markers, as detected by transcriptomic and proteomic screenings [83]. The case of rentosertib is particularly interesting since it is a potential restorative drug, leading to a slight increase in lung volume (+98 ml) over a 12-week period [64]. The successful application of biomedical AI in its development has enabled the identification of a previously overlooked IPF target, TNIK, thus opening a new direction for both anti-aging and anti-IPF interventions. Similarly, the geroprotector profile of SM04646, a Wnt inhibitor that has passed phase-1 trials, needs to be established [84, 85].

TNIK’s role in aging is yet to be fully uncovered but rentosertib’s example provides a valuable lesson for other emerging IPF research directions. The autotaxin pathway has recently been identified as a druggable IPF target, yet its involvement in aging or any geroprotective properties remain critically understudied [86]. Ziritaxestat (GLPG1690) is a novel autotaxin-targeting IPF drug candidate whose phase-3 trials were discontinued [87, 88]. This setback, however, does not mark the target as hopeless and other compounds affecting the pathway need to be explored as anti-IPF and anti-aging interventions.

Telomere attrition is another increasingly popular aging hallmark in IPF therapeutics. One of the Yamanaka factors used to rejuvenate cells, *KLF4*, has been shown to be a potential new IPF target thanks to its ability to restore *TERT* activity and prevent telomere attrition in lung epithelium [89]. In the same line, a telomerase activator extracted from the *Astragalus* herb, cycloastragenol (GRN510, TA-65), has been shown to suppress IPF-related damage in murine models [90, 91]. Cycloastragenol has also been shown to lengthen telomeres in humans, as well as counteract immunosenescence, inflammaging, lipid metabolism, and oxidative stress [92–94]. The rich set of hallmarks this compound interacts with implies its high potential to treat multiple ARDs. This hypothesis is confirmed by the motor function improvements it causes in a Parkinson’s diseases murine model [95].

As an alternative approach to restore telomerase activity, gene therapies are being tested in murine models with promising results [96]. For example, AAV9-mediated *Tert* gene therapy has improved lung function, reduced fibrosis, and alleviated inflammation in a bleomycin-induced fibrosis model [97]. In humans, danazol also represents the benefits of telomere elongation for IPF and some other ARDs, such as myelofibrosis [98, 99]. However, observational cohort studies show its lack of effect in lung forced vital capacity, a key endpoint in IPF studies [100, 101].

Based on this tight connection between aging hallmarks and IPF, we argue that a sufficiently effective anti-aging therapy has a high probability of showing clinical efficacy in IPF treatment. Thus, companies specializing in aging-oriented drug development may have a strategic advantage in designing interventions that more directly address the biological underpinnings of aging in IPF (Table 1). Conversely, the mechanisms, targets, and molecules showing promise in IPF models are also likely to have beneficial activities in a wider aging context and conditions showing a similar hallmark signature (Figure 1).

## Commercial strategy for anti-IPF drug development

In this work, we aim to provide evidence for differential alignment between ARDs and the fundamental hallmarks of aging and highlight the importance of the hallmark framework in clinical research. Our analysis demonstrates that certain ARDs more closely reflect the cellular aging processes, making them ideal candidates for testing geroprotective interventions. IPF emerges as an emblematic aging disease due to its strong representation across multiple aging hallmarks and promising findings in clinical programs.

We argue that the etiology of IPF fundamentally mirrors that of aging itself, with multiple hallmarks interacting synergistically to drive disease progression. This claim is backed both by our computational approach and literature evidence of anti-aging interventions counteracting IPF [72, 74]. This close alignment makes IPF a perfect testbed for anti-aging therapeutics, as insights gained from treating it will undoubtedly inform our approach to aging and numerous downstream ARDs.

**Figure 3:**
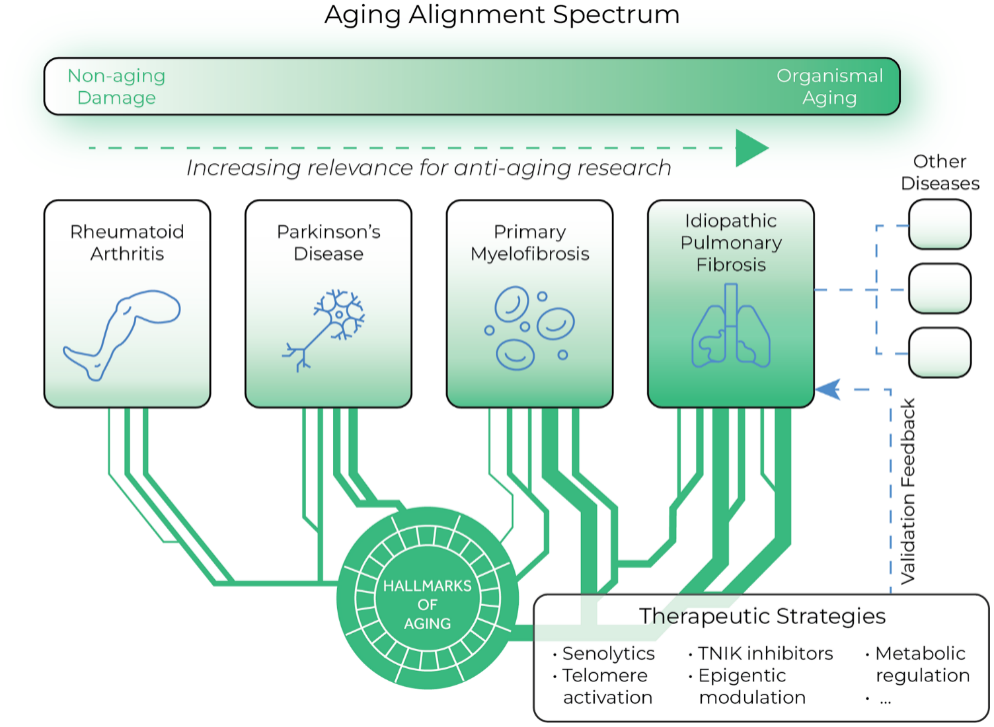
**Conceptual framework for aging alignment in disease models and therapeutic development.** This schematic illustrates the spectrum of aging-related diseases (ARDs) based on their mechanistic overlap with fundamental aging processes. Based on our assessment, idiopathic pulmonary fibrosis (IPF) demonstrates the strongest aging alignment. This framework supports a bidirectional approach to therapeutic development: insights from aging biology inform disease-specific interventions, while clinical efficacy in high-alignment conditions like IPF provides validation feedback for potential geroprotective strategies. The therapeutic strategies shown (senolytics, telomere activation, TNIK inhibitors, epigenetic modulation, and metabolic regulation) represent promising approaches that may translate across multiple ARDs based on their promising results in IPF models and human patients.

A key implication of this work suggests that interventions potent enough to address core aging processes should demonstrate efficacy across multiple ARDs. We propose that pharmaceutical development should incorporate hallmark analysis when selecting indications, molecular targets, and compounds to maximize translational potential (Figure 3). This approach may significantly reduce development timelines and increase success rates.

From a strategic perspective, developing an anti-aging therapy for IPF offers major opportunities for indication expansion. Compared to the currently dominant approach of single disease trials, targeting aging mechanisms may prove a strictly better strategy. The promising therapeutics explored in IPF (senolytic combinations, telomerase activators, and epigenetic modulators) represent approaches that may generalize across the spectrum of ARDs. Following a successful trial in IPF, such therapies could be extended to other fibrotic or aging-related conditions including liver cirrhosis, chronic kidney disease, atherosclerosis, and many others sharing the underlying aging mechanisms [24]. The unification of multiple ARDs under the umbrella of hallmark-driven diseases offers the convenience of more informed, and thus, more cost- and time-efficient drug development pipelines (Table 2). Recent analyses demonstrate that pharmaceutical development costs range from $172-515 million per drug when accounting for direct costs, rising to nearly $900 million when including capital costs and failures [102–104]. Notably, phase-3 trials, which constitute the largest portion of this expenditure at $100-300+ million per trial, could potentially be streamlined through targeting common aging mechanisms applicable across multiple diseases, rather than conducting separate trials for each age-related condition. But most importantly, the success in IPF could then accelerate the legislation of anti-aging trials and attract much needed investment to the field of biogerontology, thus transforming the whole landscape of aging research.

**Table 2:** Estimated drug development timeline and costs for IPF.

**Stage**	**Duration (Years)**	**Estimated cost (USD)**	**Notes**
Target Discovery and Validation	Traditional: 1–3 years; AI-driven: <1 year	~$1–10 million	AI can compress this phase
Hit Discovery and Lead Optimization	Traditional: 2–4 years; AI-driven: 1–2 years	~$10–50 million (small molecules); up to ~$100 million with full preclinical work	AI generative design accelerated discovery to candidate in ~18 months
Preclinical Development	1–2 years	~$5–15 million	Includes Investigational New Drug enabling studies (Safety and Toxicity; Pharmacokinetics; Chemistry, manufacturing, and controls)
Phase I Clinical (Safety)	~1 year	~$5–10 million	Typically involves 50–100 subjects; IPF studies may use healthy volunteers or patients
Phase II Clinical (Efficacy)	~1–2 years	~$20–50 million	100–200 IPF patients; shorter duration than chronic diseases due to rapid progression
Phase III Clinical (Pivotal)	2–3 years (often 1-year treatment per trial)	~$100–300+ million (per trial)	Larger global trials with 500–800 patients; orphan status may reduce numbers but not costs
Regulatory Review and Approval	0.5–1.5 years	~$2–5 million	Orphan drug fees may be waived; accelerated review is possible
Post-market Surveillance	Ongoing	Variable ($10–50+ million)	Part of life-cycle management

The table summarizes the typical duration, estimated costs, and key considerations across the drug development pipeline for IPF therapies. Cost estimates are derived from [102–104] and adjusted to reflect the orphan status of IPF. AI-driven approaches can significantly compress early development phases compared to traditional methods. The high costs of later-stage clinical trials highlight the potential efficiency gains of targeting common aging mechanisms shared by multiple ARDs rather than conducting separate development programs for each indication.

To promote the shift toward hallmark-aware drug development, companies conducting clinical trials should incorporate ancillary analytics of aging biomarkers in their studies, as they may be overlooking broader anti-aging effects of their therapies. For this purpose, aging clocks serve as a perfect tool thanks to a wide range of supported data types, interpretability, a long validation record, and permissive licenses [105–109].

In conclusion, our hallmark-based analysis and literature review provide a framework for identifying optimal disease models for geroprotector discovery. IPF offers a gateway to broader therapeutic applications across multiple ARDs, supporting the view that targeting aging itself represents an efficient drug design strategy.

## METHODS

**Figure 4:**
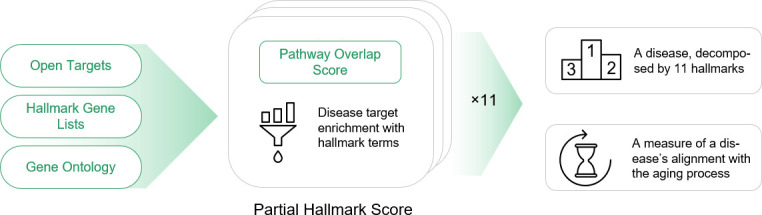
A brief overview of the scoring methodology used to measure a disease's alignment with the general aging process.

Our study utilized a custom approach to analyze and compare ARDs by integrating data from multiple sources (see Figure 4). Disease-associated target genes were acquired through the Open Targets Platform [110], which aggregates evidence of target-disease associations from scientific literature. Only high-evidence (target score>0.4) genes were considered in the enrichment analysis. Out of the genes passing the threshold, we randomly selected 50 out of top-60 to calculate the partial hallmark scores and the total scores. This procedure was repeated 250 times for each disease to get 95% confidence intervals of the scores and to derive statistical significance of non-zero hallmark scores. The distributions of partial hallmark and total scores are available in Supplementary Material 1. For partial scores on Figure 1, q-values were calculated with Benjamin-Hochberg correction to test the alternative hypothesis that the true hallmark score is greater than zero. All scores presented in the figures are normalized relative to the maximal score achieved in sampling.

For pathway enrichment analysis, we employed the GO:BP ontology accessed via Enrichr [111]. Each pathway in the GO:BP was annotated as belonging to one or more aging hallmarks. Pathway hallmark assignment was carried out by AI agents enabled with access to GPT-4 and tools providing access to GO:BP and NCBI to increase annotation relevance.

The list of hallmarks to map the diseases to is obtained from [7], except dysbiosis due to its dependence on non-human targets. We then defined a metric of a disease’s alignment with each of the eleven selected hallmarks based on its targets’ enriched pathways.

Pathway scores *P_h,d_* were derived from pathway enrichment analysis (as implemented in Enrichr), with all significantly enriched pathways (p-value<0.001) contributing to the corresponding hallmark partial score. For a set of enriched pathways *E_d_*, the pathway score was calculated as:


$$\begin{array}{l} P_{h,d}=\sum_{p\in R_{h,d}}[-\log_{10}(p\text{-}value)]\times N_{h}\\ \;\;\;\;\;\;\;\;\;\;\;\;\;\;\;\;\times \left(1+\frac{\log_{2}\left(\left|R_{h,d}\right|+1\right)}{4}\right) \end{array}$$

where *R_h,d_* is the subset of pathways in *E_d_* relevant to hallmark *h*, *p-value* is the enrichment p-value of pathway *p*, and *N_h_* is a normalization factor accounting for the uneven distribution of pathways across different hallmarks.

In the current version the weights are selected arbitrarily, with a lower weight assigned to the gene list component due to lower annotation density in the used gene lists.

Finally, the overall aging score *A_d_* for disease *d* was calculated as:


$$A_{d}=\sum_{h\;\in \;H}P_{h,d}\times D_{f}$$

where *H* is the set of all hallmarks and *D_f_* is a diversity factor that rewards diseases with multiple hallmark associations rather than a single dominant one:


$$D_{f}=\frac{N_{nz}}{N_{t}}\times (1+E_{n})$$

with *N_nz_* being the number of non-zero hallmark scores, *N_t_* the total number of hallmarks (11), and *E_n_* the normalized entropy of the hallmark score distribution.

The final metric *A_d_* accounts for both the magnitude and distribution of partial hallmark scores *T_h,d_*, thus providing a quantitative basis for identifying the similarities in the aging-related mechanisms underlying different diseases.

The code for this disease scoring method is written in Python 3.11 and is available on Github with demonstration materials: https://github.com/Insilico-org/disease_hallmarks

The proposed methodology is intended as an illustration of the proposed hallmark decomposition approach to geroprotector and drug discovery. In the current form it holds several limitations that need to be elaborated on in later iterations. The scoring depends on the available information about target proteins and thus may undervalue the importance of understudied hallmarks. Similarly, gene annotation density and the sources of these annotations may bring in certain biases.

## Supplementary Material

Supplementary Material
