# Supplementary material for: Age-related diseases as a testbed for anti-aging therapeutics: the case of idiopathic pulmonary fibrosis
Source: Aging (Albany NY). 2025 Aug 16;17(8):1911–28. doi: 10.18632/aging.206301 (PMC12422794; doi:10.18632/aging.206301)

# **Supplementary Material 1. Visualizations of partial hallmark and total aging score distributions for the 13 aging-related diseases obtained in sampling.**

# Age-related Diseases as a Testbed for Anti-aging Therapeutics: The Case of Idiopathic Pulmonary Fibrosis

## Authors

Alex Zhavoronkov^1,2,3, †^, Dominika Wilczok^4^, Feng Ren^1,2,3,5^, Fedor Galkin^1^

## Affiliations

1 — Insilico Medicine AI Limited, Abu Dhabi, UAE

2 — Insilico Medicine US, Inc., Boston, MA, USA

3 — Insilico Medicine Hong Kong Ltd., Hong Kong Science and Technology Park, Hong Kong SAR, China

4 — Duke University, Durham, NC 27708, USA

5 — Insilico Medicine Shanghai Ltd., Shanghai, China

† — Corresponding author ([alex@insilico.com](mailto:alex@insilico.com))

## Supplementary Material 1

This file contains visualizations of pre-normalization resampled scores for all 14 ARDs presented in Figures 1-2 of the main text. For reproduction purposes, all these visualizations may be obtained from the demonstration notebook deposited at the accompanying [GitHub repository](https://github.com/Insilico-org/disease_hallmarks).

In the following visualizations, the red dashed line represents the mean scores (partial or total) obtained from 250 resamplings. All images obtained with Plotly v5.23.0 for Python v3.11


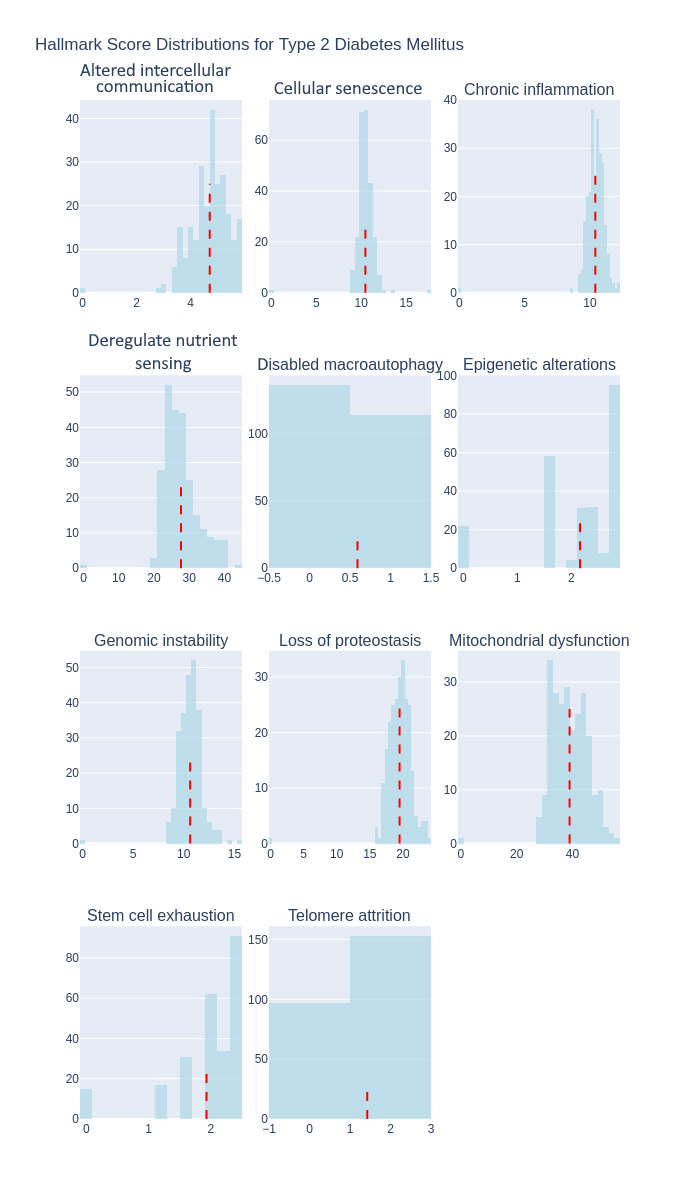

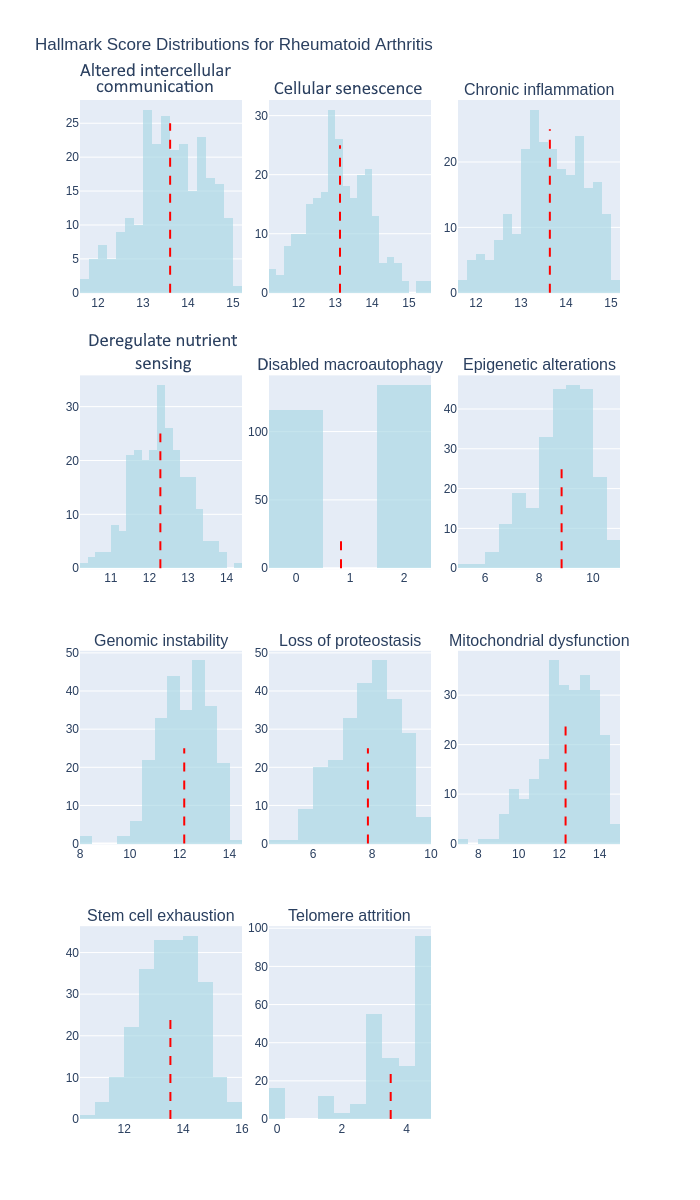

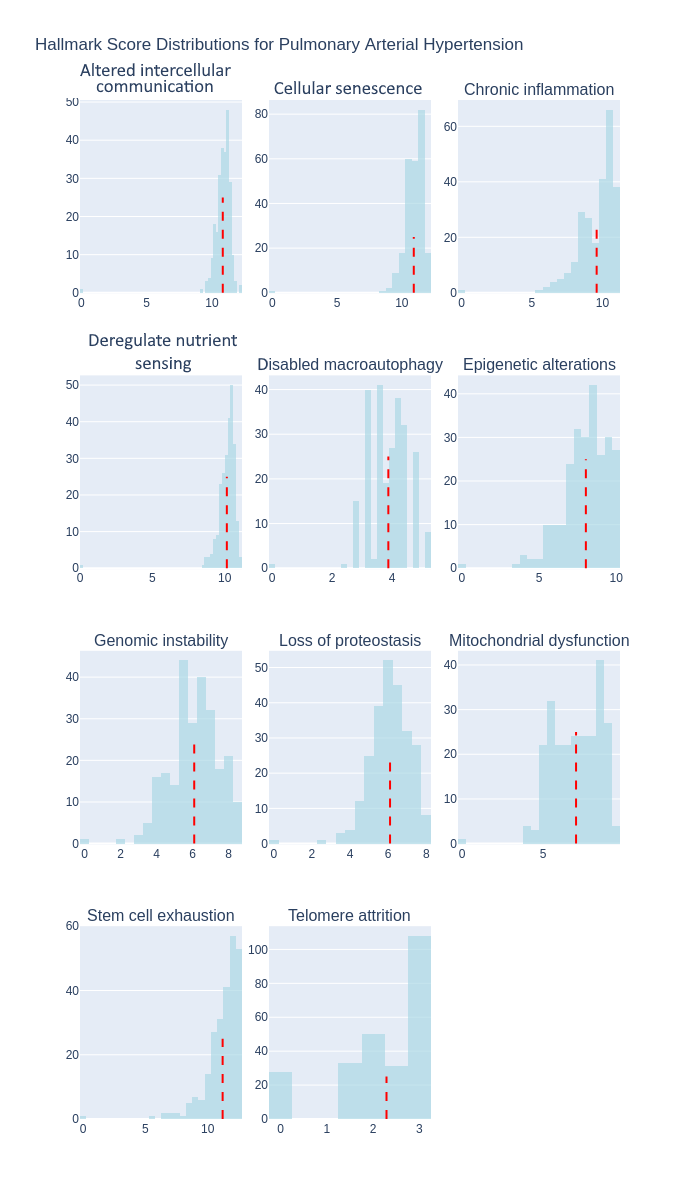

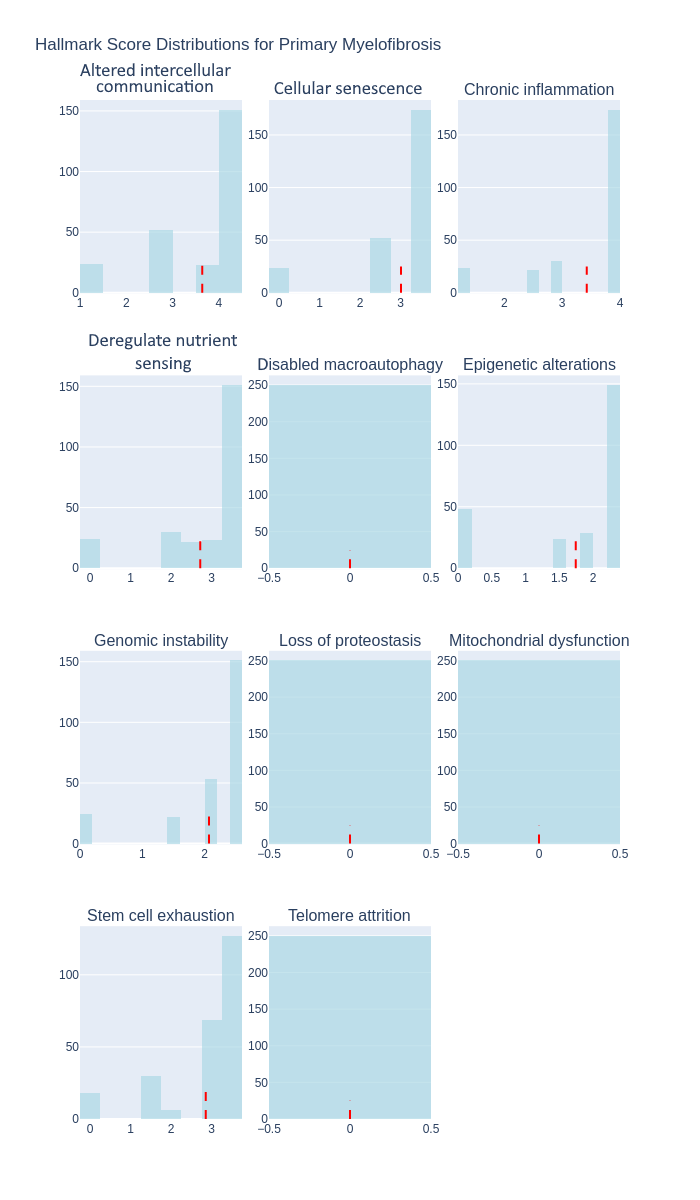

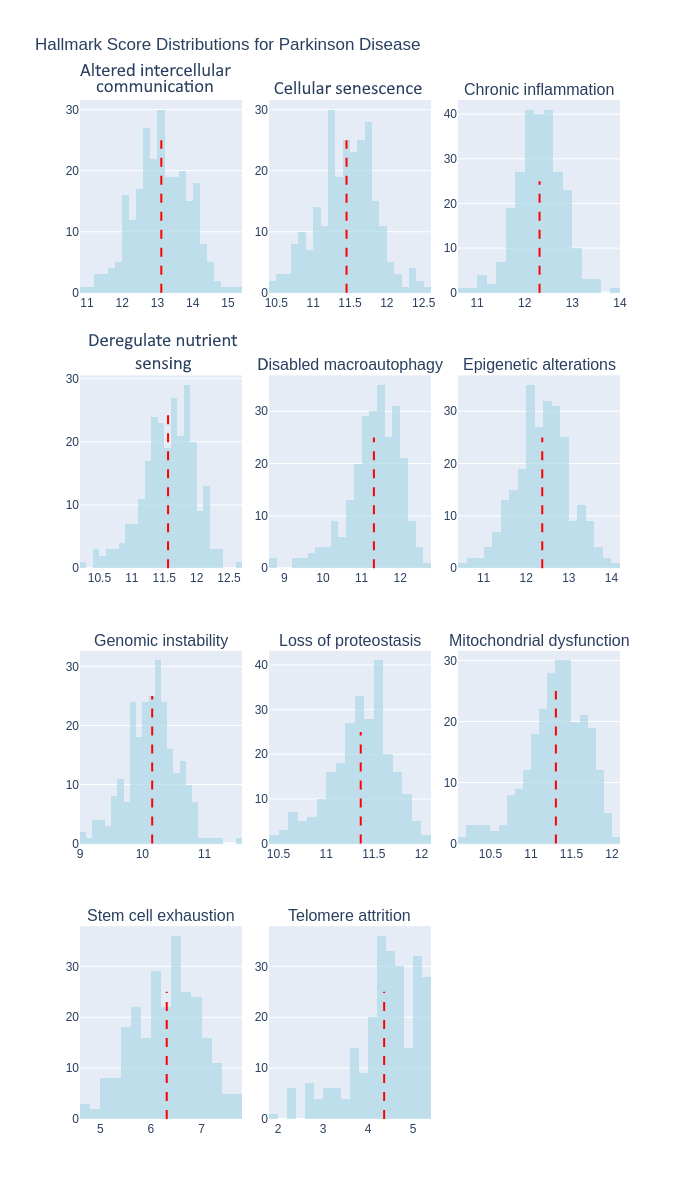

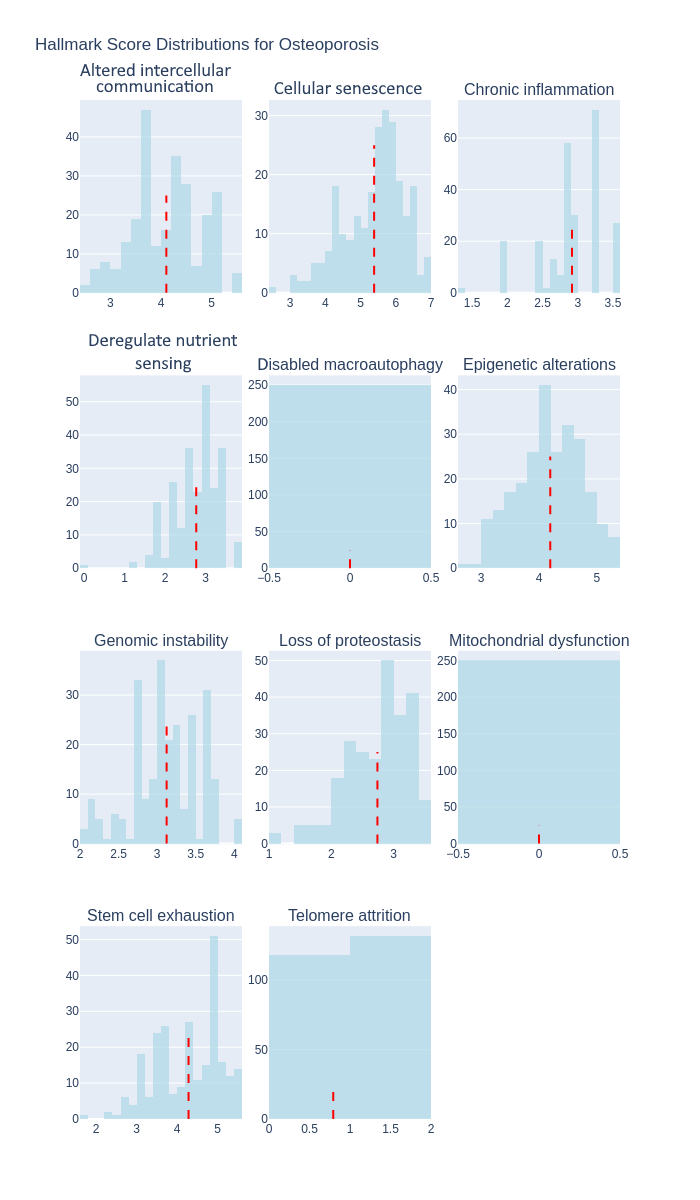

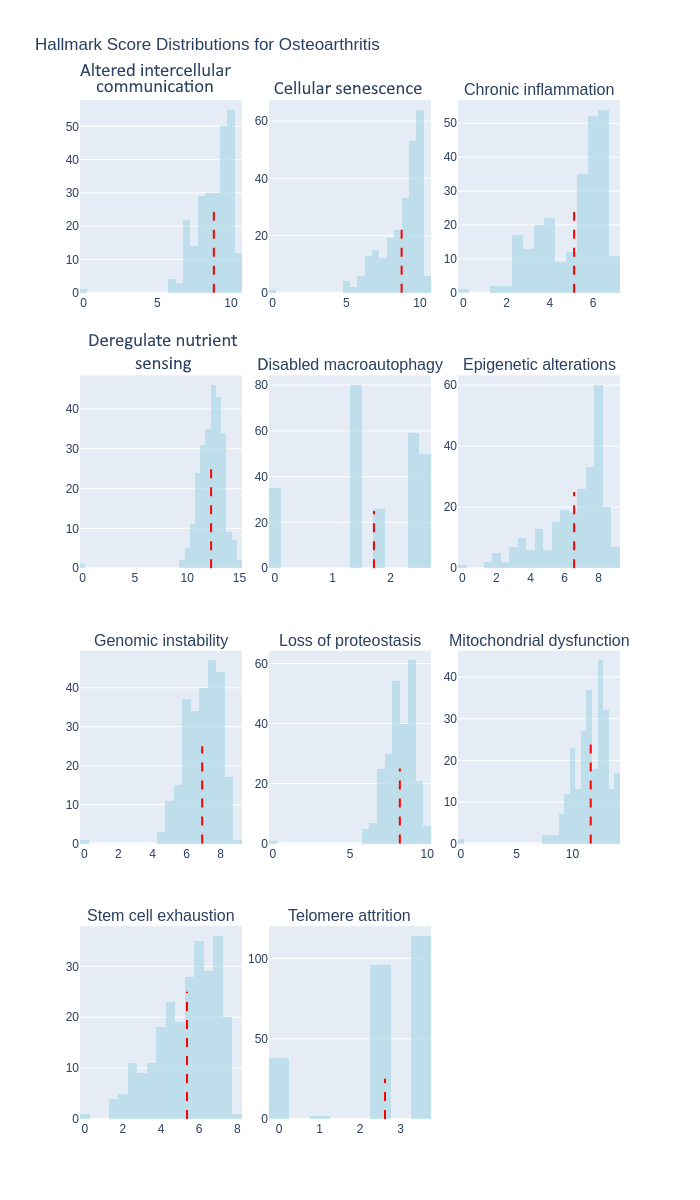

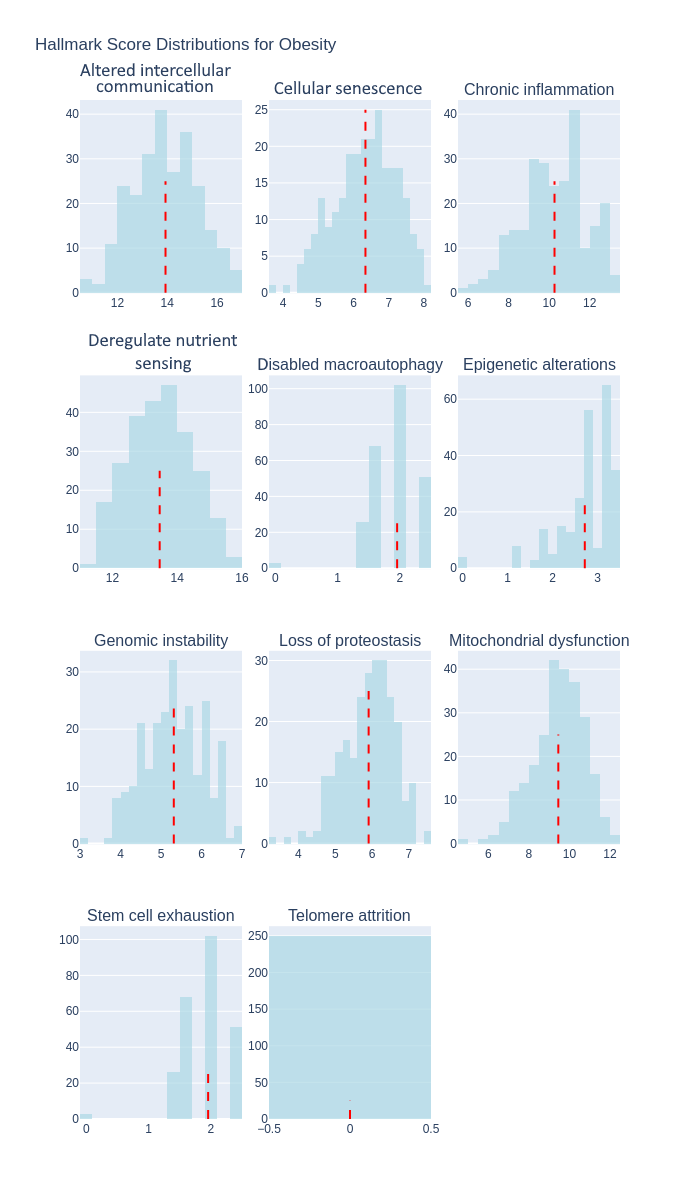

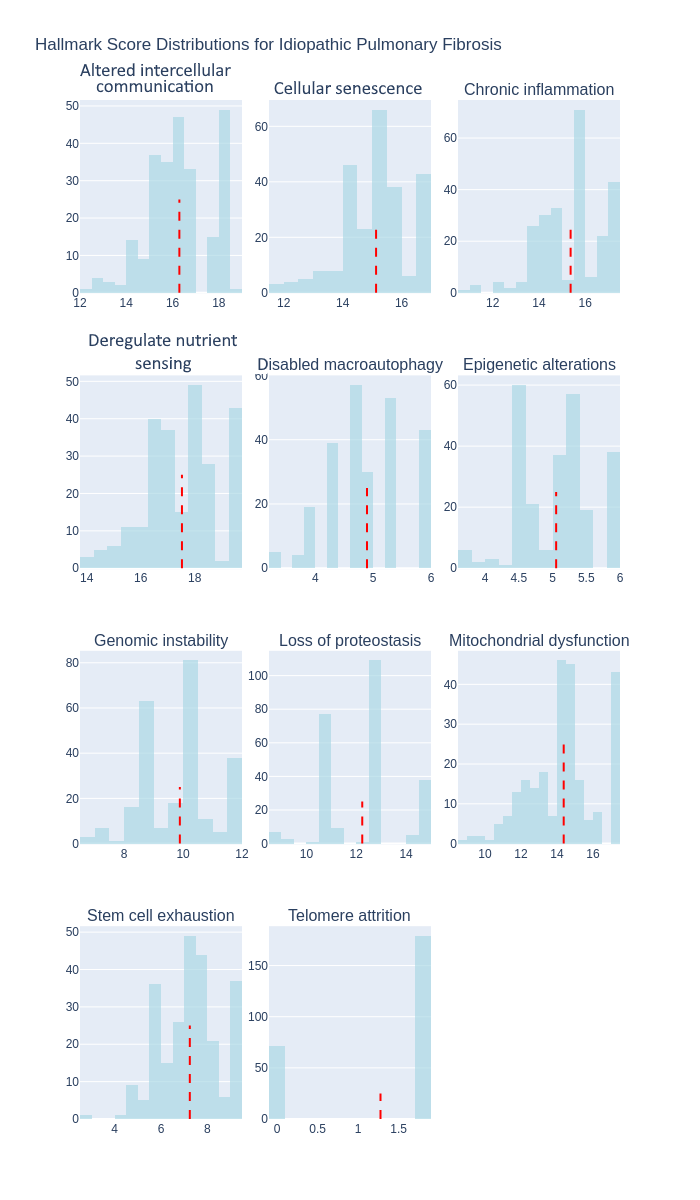

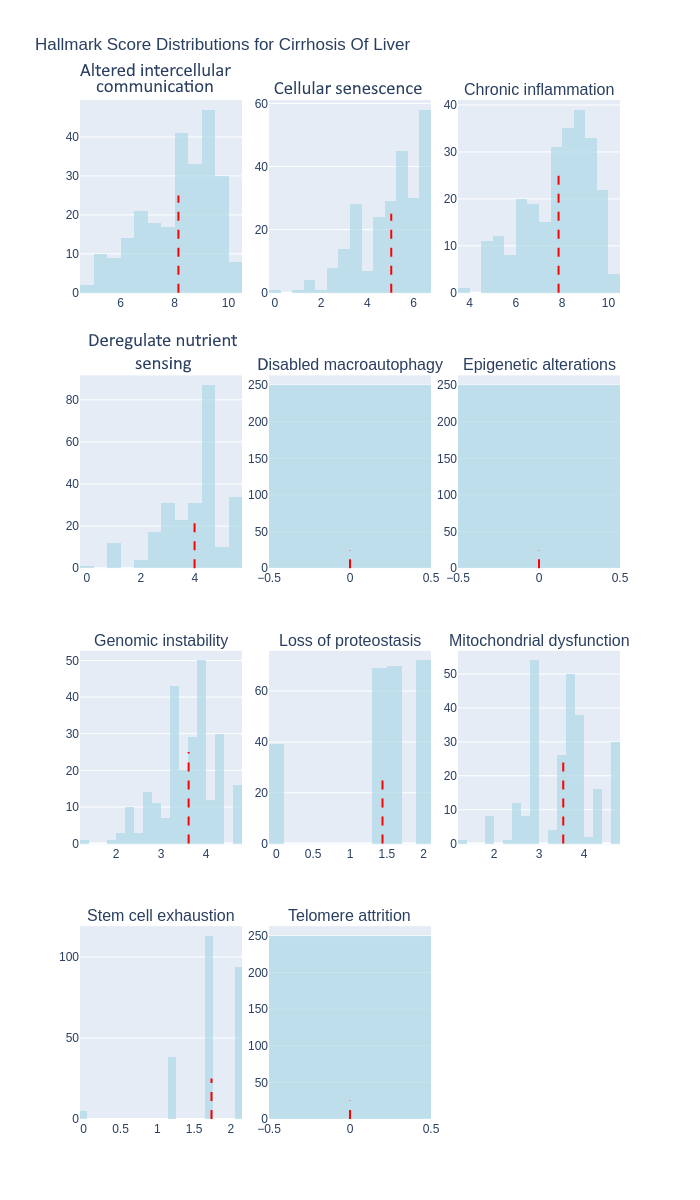

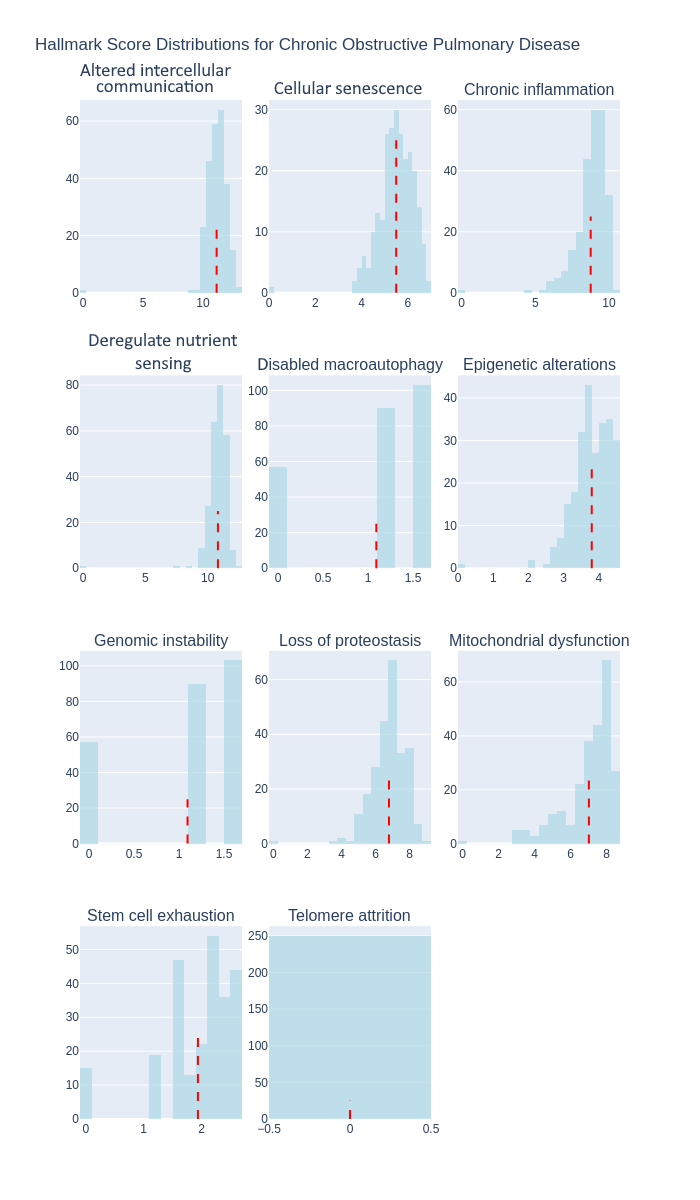

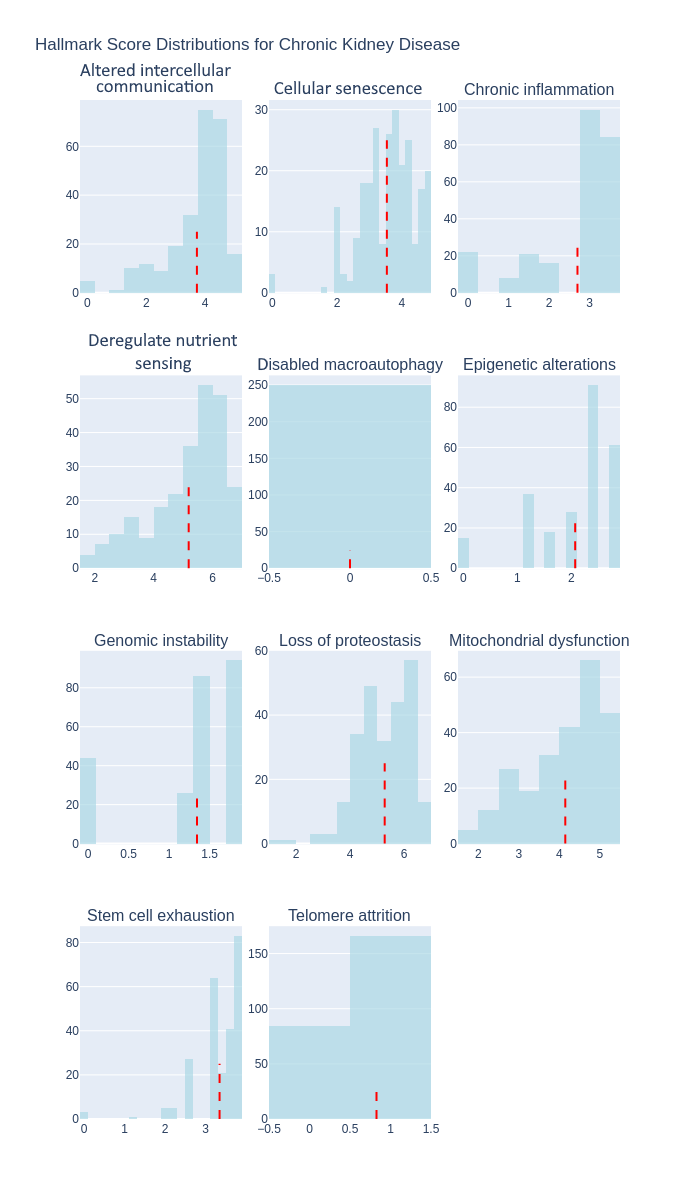

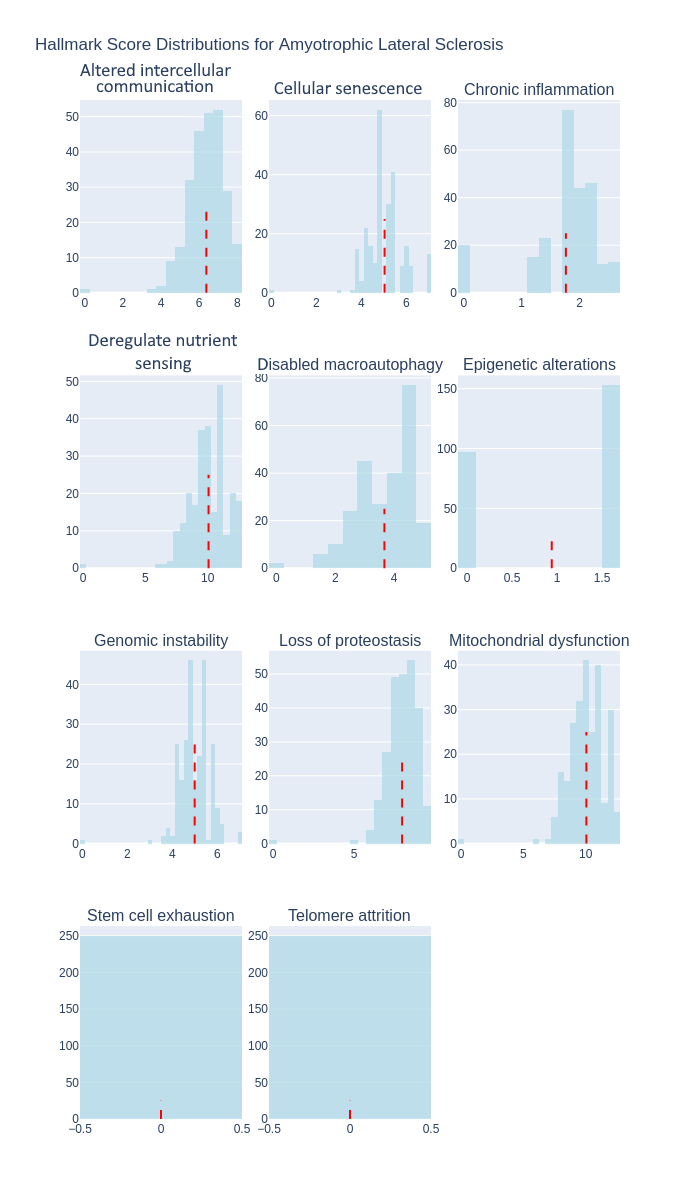


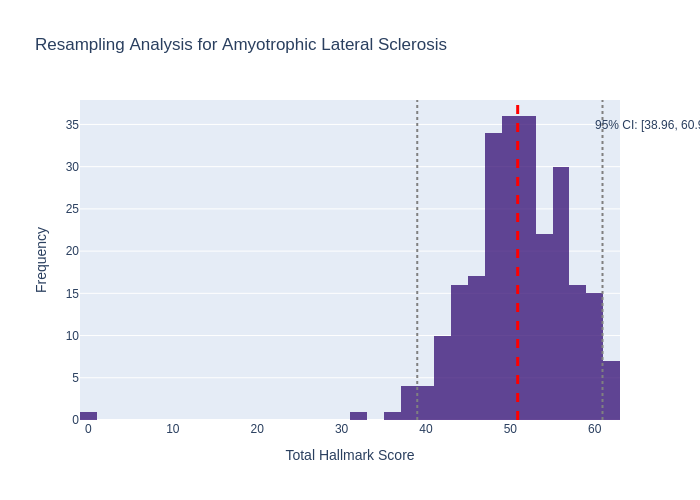

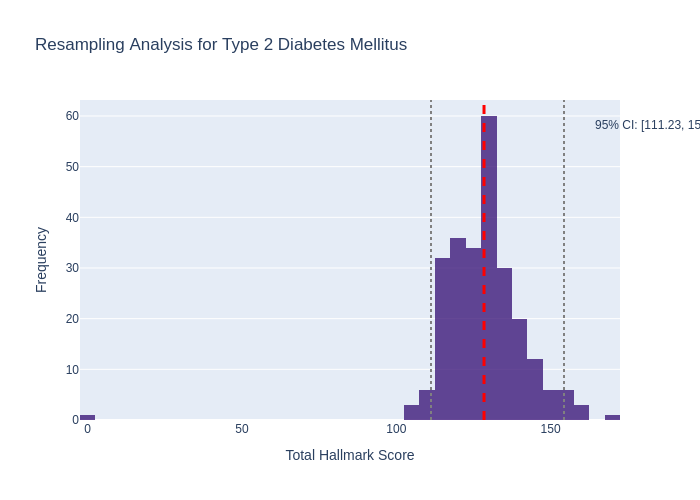

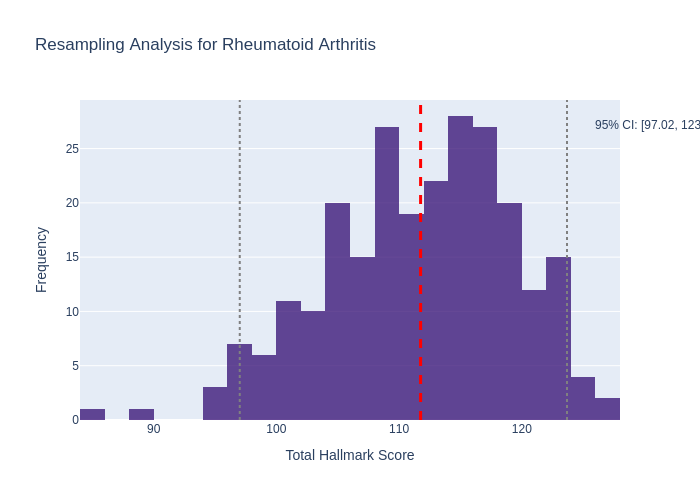

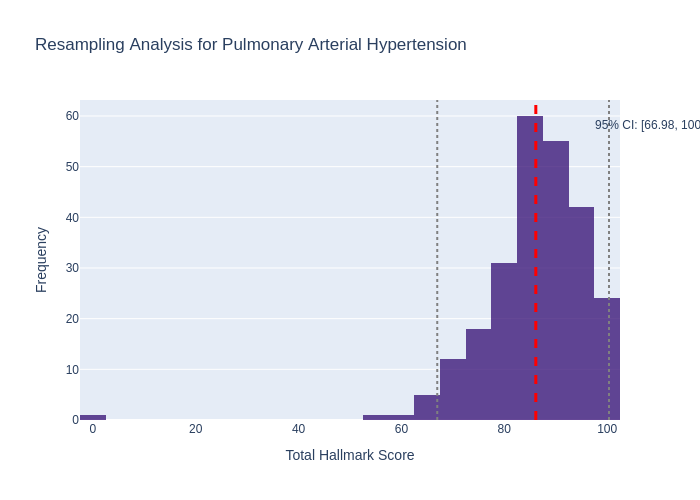

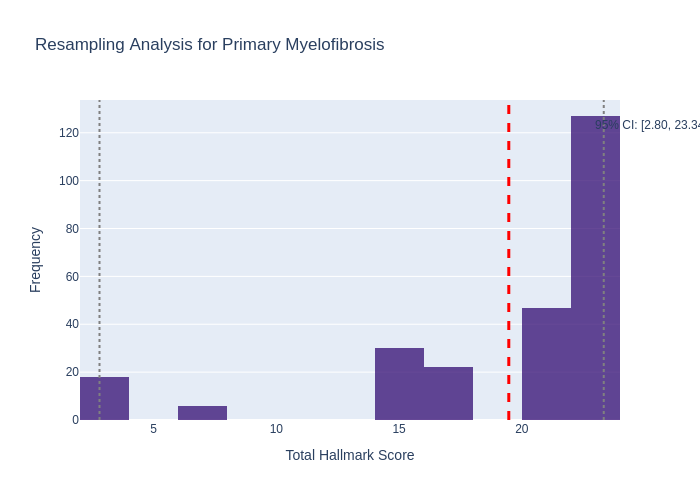

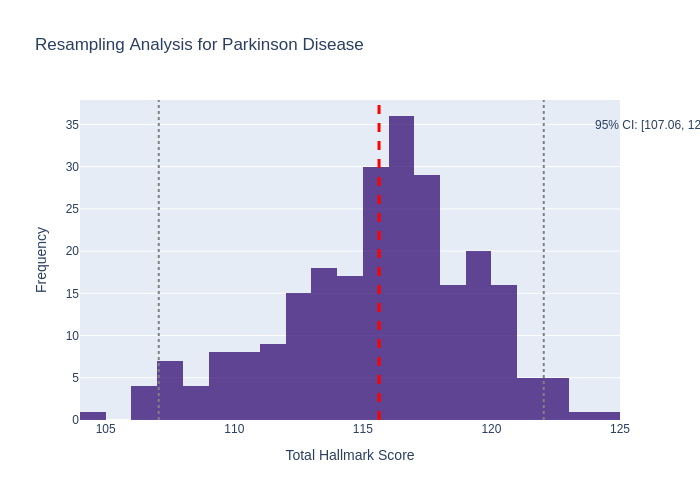

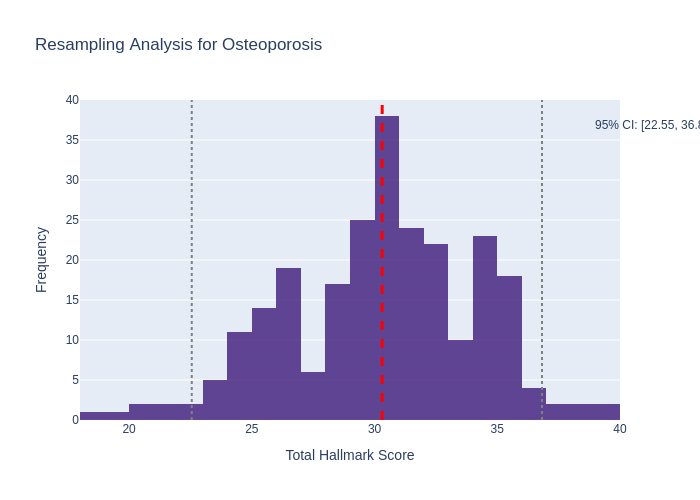

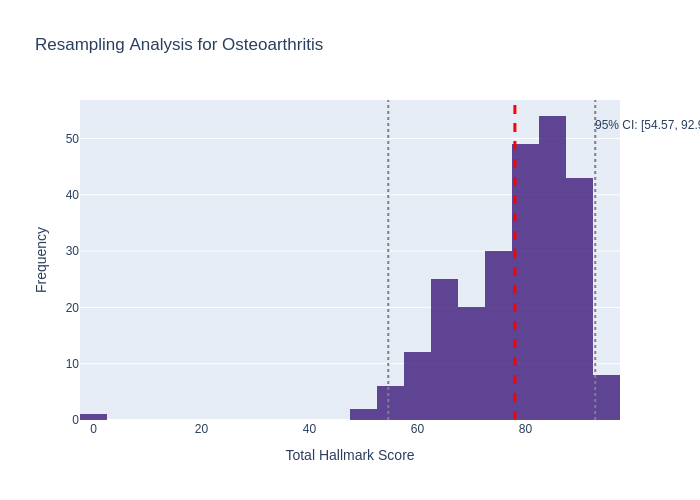

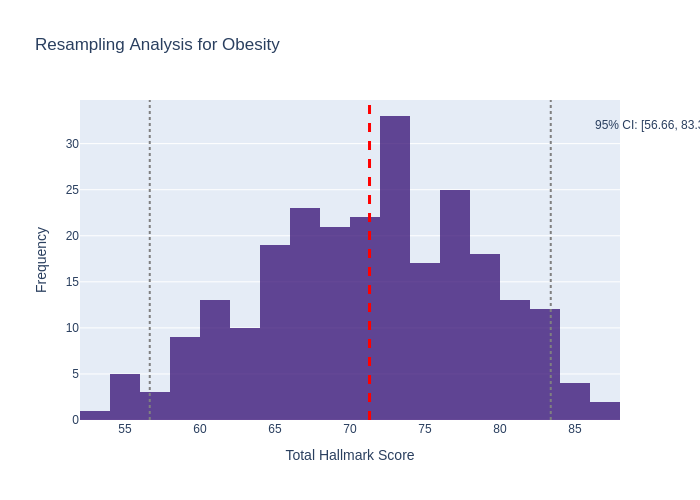

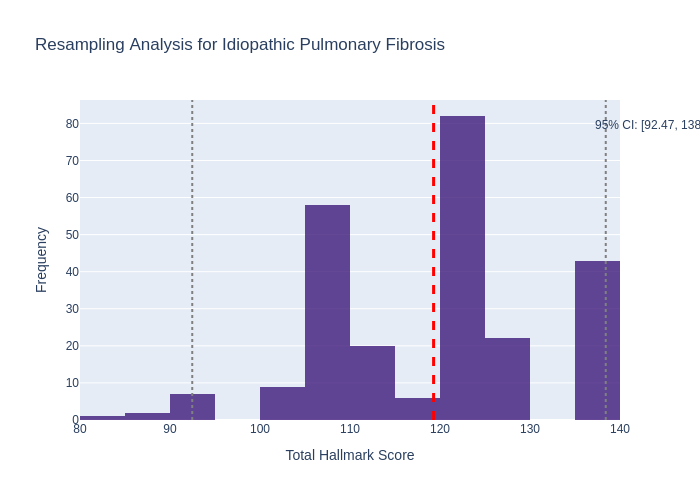

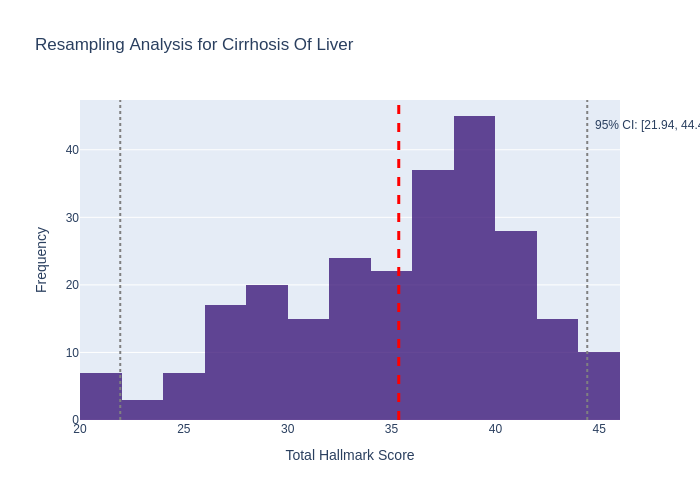

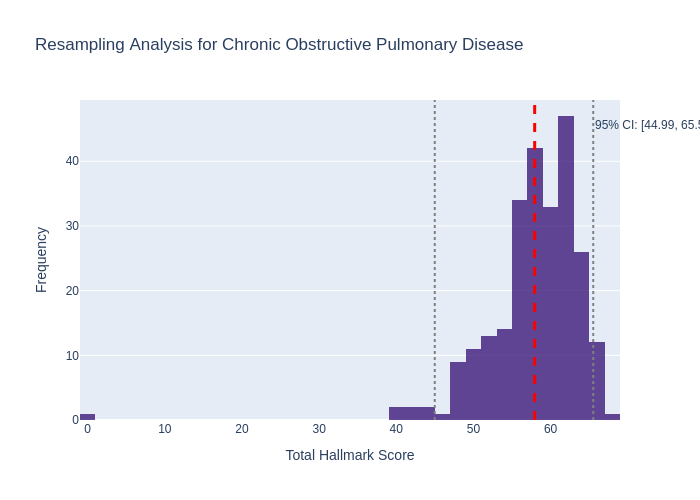

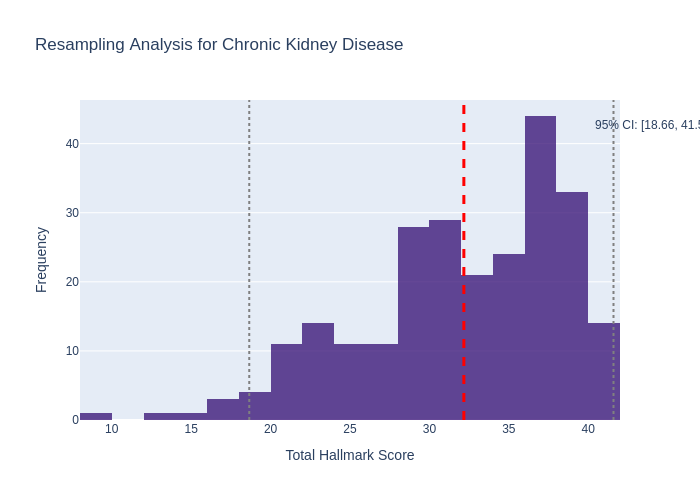

Supplement: Supplementary Material [file aging-17-8-206301-s001.docx]
